# Dopamine D_2_-Receptor Antagonists Down-Regulate CYP1A1/2 and CYP1B1 in the Rat Liver

**DOI:** 10.1371/journal.pone.0128708

**Published:** 2015-10-14

**Authors:** P. Harkitis, E. P. Daskalopoulos, F. Malliou, M. A. Lang, M. Marselos, A. Fotopoulos, G. Albucharali, M. Konstandi

**Affiliations:** 1 Department of Pharmacology, Faculty of Medicine, University of Ioannina, Ioannina GR-451 10, Greece; 2 University of Queensland, National Research Centre for Environmental Toxicology (Entox), 39 Kessels Road, Coopers Plains, QLD 4108, Australia; 3 Department of Nuclear Medicine, Faculty of Medicine, University of Ioannina, Ioannina GR-451 10, Greece; Nihon University School of Medicine, JAPAN

## Abstract

Dopaminergic systems regulate the release of several hormones including growth hormone (GH), thyroid hormones, insulin, glucocorticoids and prolactin (PRL) that play significant roles in the regulation of various Cytochrome P450 (CYP) enzymes. The present study investigated the role of dopamine D_2_-receptor-linked pathways in the regulation of CYP1A1, CYP1A2 and CYP1B1 that belong to a battery of genes controlled by the Aryl Hydrocarbon Receptor (AhR) and play a crucial role in the metabolism and toxicity of numerous environmental toxicants. Inhibition of dopamine D_2_-receptors with sulpiride (SULP) significantly repressed the constitutive and benzo[a]pyrene (B[a]P)-induced *CYP1A1*, *CYP1A2* and *CYP1B* expression in the rat liver. The expression of *AhR*, heat shock protein 90 (*HSP90)* and AhR nuclear translocator *(ARNT)* was suppressed by SULP in B[a]P-treated livers, whereas the *AhRR* expression was increased by the drug suggesting that the SULP-mediated repression of the CYP1 inducibility is due to inactivation of the AhR regulatory system. At signal transduction level, the D_2_-mediated down-regulation of constitutive *CYP1A1/2* and *CYP1B1* expression appears to be mediated by activation of the insulin/PI3K/AKT pathway. PRL-linked pathways exerting a negative control on various CYPs, and inactivation of the glucocorticoid-linked pathways that positively control the AhR-regulated CYP1 *genes*, may also participate in the SULP-mediated repression of both, the constitutive and induced *CYP1* expression. The present findings indicate that drugs acting as D_2_-dopamine receptor antagonists can modify several hormone systems that regulate the expression of *CYP1A1*, *CYP1A2* and *CYP1B1*, and may affect the toxicity and carcinogenicity outcome of numerous toxicants and pre-carcinogenic substances. Therefore, these drugs could be considered as a part of the strategy to reduce the risk of exposure to environmental pollutants and pre-carcinogens.

## Introduction

*CYP1A1*, *CYP1A2* and *CYP1B1* belong to a battery of *genes* that are transcriptionally activated by the aromatic hydrocarbon receptor [1]. More than 90% of known chemical carcinogens, including aromatic amines and polycyclic aromatic hydrocarbons (PAH)s, are substrates of these cytochromes [2–8], and their metabolism often results in the formation of active carcinogenic metabolites [9,10]. Benzo[a]pyrene (B[a]P) is the major PAH component in cigarette smoke and environmental mixtures, such as coal tar and diesel exhaust condensate and is found in the heavily polluted air of urban and industrial areas, in water and heavily cooked food [11]. B[a]P is partly metabolized by CYP1A isozymes to an electrophilic reactive intermediate that covalently binds to DNA and initiates carcinogenesis [3,5]. In addition, B[a]P, acts as a ligand of the AhR and as an inducer of the CYP1 enzymes. The dual role of B[a]P as an inducer of CYP1A1/2 and CYP1B1 and as a pre-carcinogenic substrate for these cytochromes, indicates that B[a]P and related compounds constitute a particularly important group of toxicants able to enhance their own metabolic activation and carcinogenicity [12].

Previous studies have shown that psychological stress and adrenergic receptor (AR)-linked pathways can regulate the expression of cytochrome P450 enzymes [13–18]. Specifically, restraint stress up-regulated *CYP1A2* in the murine and rat liver [13,19,20], and AR-agonists or antagonists, and drugs modifying central and peripheral catecholaminergic activity, have a strong impact on the expression of constitutive and B[a]P-induced *CYP1A1/2* expression [13]. These results suggest a strong regulatory role of stress and related adrenergic signalling pathways in the regulation of both constitutive and B[a]P induced CYP1A1/2 expression [13,21].

Dopaminergic systems play also significant roles in the regulation of several CYP isozymes catalyzing the metabolism of the majority of prescribed drugs [21–23]. In particular, inhibition of dopamine D_2_-receptors markedly repressed hepatic *CYP2C11*, *CYP2D1/2*, *CYP2E1* and *CYP3A1/2* expression in rats [22,23]. In this regulatory loop the role of insulin/PI3K/AKT signalling pathway is critical [24].

The D_2_-dopaminergic receptor-mediated CYP regulation is potentially highly significant as a wide array of drugs, prescribed for a variety of diseases, such as psychosis, depression, bipolar disorder and Parkinson's disease, exert their effects mainly via D_2_-dopaminergic receptor-linked pathways [25]. These drugs acting as either D_2_-receptor-agonists or antagonists can modify the activity of several hormonal pathways including the insulin/PI3K/AKT signalling pathway thus influencing the expression of various drug metabolizing cytochromes. This effect may lead to significant drug-drug interactions and may influence the outcome of pharmacotherapy and drug toxicity [18,26,27].

The aim of this study was to investigate the role of D_2_-dopaminergic receptor- related pathways in the regulation of cytochrome CYP1A1, CYP1A2 and CYP1B1 in the liver. For this purpose, rats were treated with selective D_2_-antagonists and exposed to either B[a]P or the vehicle alone [22]. The findings indicated the critical role of dopamine D_2_-receptors in the regulation of the constitutive and B[a]P-induced expression of these cytochromes, and suggest that drugs binding to dopamine D_2_-receptors may modify the toxicity of environmental pollutants and pre-carcinogens interfering with their metabolism.

## Materials and Methods

### Animals

Adult male inbred Wistar rats (Kuo/Ioa/rr) 3 months old (weighing 250–300g) were used for this study. All animals were housed in groups of 5 and maintained in plastic cages (Makrolon) with wood chip bedding, under a constant temperature (20°C) and a 12h light/dark cycle. Food (the standard rodent chow) and tap water was available *ad libitum*. All *in vivo* animal experiments and *in vitro* experiments employing primary hepatocytes isolated from rats were reviewed and approved by the Institutional Animal Care and Use Committee of the Medical School of the University of Ioannina, and the study has been carried out in strict accordance with the recommendations in the National Institutes of Health Guide for the Care and Use of Laboratory Animals (NIH Publications No. 80–23), revised 1996 and with the Guiding Principles in the Use of Animals in Toxicology, the adopted by the Society of Toxicology in 1989. Efforts were made in order to minimize the number of animals used and reduce their suffering.

### Drugs

The following drugs have been used in this study: Sulpiride (Sigma, USA.); benzo[a]pyrene (Sigma- Aldrich, USA) and L-741,626 (SID 50104688 in PubChem; Sigma-Aldrich, USA).

### Experimental procedure

Groups of five animals each received the selective dopamine D_2_-receptor antagonist, sulpiride (12mg/kg b.w., s.c.; SULP) or the vehicle (normal saline). Alternatively, the highly selective dopamine D_2_-receptor antagonist, L-741,626 (1.5mg/kg b.w., i.p.) was also administered in a group of rats. The drugs were administered twice daily and for four consecutive days (totally 7 injections in each treatment group). In parallel to SULP treatment, the animals received either olive oil or benzo[a]pyrene (10mg/kg b.w., i.p.) once daily for three consecutive days. The last dose of B[a]P was administered 24 hours before sacrifice.

At the end of the experiment, two hours after the last drug treatment, the animals were sacrificed by decapitation and trunk blood was collected for hormonal determinations. The samples were kept at -20°C until assayed. Simultaneously, the brains were rapidly removed and the dissected hypothalamus was immediately frozen in liquid nitrogen and stored at -80°C until assayed. Parts of the livers were also taken for microsome isolation, total RNA, nuclear and cytosol extraction and were kept at -80°C until analyzed.

### Neurochemical analysis

Dopaminergic activity in the hypothalamus was assessed by measuring DA, DOPAC and HVA concentrations. Hypothalamic DA turnover ratio was determined by calculating the rates of DOPAC over DA levels in this brain region. The DOPAC/DA turnover ratio was used as an index of the DA turnover rate, which reflects the dopaminergic activity including the release and/or metabolism of DA, because evidence suggests that dopaminergic activity is better evaluated with DOPAC/DA than with tissue DA, DOPAC and HVA levels [28,29]. For the determination of DA and its metabolites concentration in the hypothalamus, the high performance liquid chromatography with electrochemical detection (HPLC-EC) was used, as previously described [30–32], with some minor modifications. Briefly, the samples were weighed and homogenized for 20 s with a sonicator in ice cold 0.2 N perchloric acid (HClO4). The homogenate was centrifuged at 13,000 rpm at 4°C for 15 min and the supernatant was divided into two portions. The measured compounds were DOPAC and HVA. An aliquot of 200 μl was transferred to an eppendorf tube containing 20 mg activated alumina and was used to extract dopamine (DA) and DOPAC from the homogenate prior to HPLC detection. The following operating conditions were used: column type: Apex-C-18 ODS 5μ (Jones) reverse phase column; mobile phase: 0.1 M sodium acetate (CH3-COONa), 0.1 M citric acid (C6H8O7.H_2_O), 2.7 x 10^−4^ M octyl sulphate (C8H17O4; Sna), with 25% methanol (v/v); flowrate: 0.6 ml/min; detector: electrochemical detector (Shimadzu, Japan) maintained at 0.75 V. Data were acquired on a PC-compatible computer using BAS-5 interface. Standard curves were constructed using 8 points between 0.625 and 80 pg/10 μl for dopamine, 23.4–3000 pg/10 μl for DOPAC and 15.62–2000 pg/10 μl for HVA. Correlation coefficients (r) of >0.98 were obtained for all curves. The working standard solutions were kept at -80°C and 10 μl of the standard solution was injected in the beginning of the analysis and between biological samples.

### Isolation of microsomes

Microsomal fractions were prepared by homogenization of rat liver samples in ice-cold homogenization buffer (0.15 M KCl, 10 mM K_2_EDTA, 1mM Dithiothreitol, pH 7.4). The homogenates were centrifuged at 9,000 g (4°C) for 20 min. The upper phase was transferred carefully into new vials and was centrifuged for 70 min at 96,552 g (4°C). After removal of the liquid phase, followed washing of the microsomal pellet: re-suspension in ice-cold homogenization buffer, homogenization and centrifugation for 60 min at 96,552 g (4°C). The washed microsomal pellet was re-suspended in ice-cold storage buffer (K_2_HPO4/KH_2_PO4 pH 7.4, 1 mM K_2_EDTA, 0.1 mM dithiothreitol, 20% glycerol) and stored at -80°C until assayed [33].

### Assessment of hepatic EROD and MROD activities

In the microsomes of rat livers the CYP1A1/2-dependent activities were determined. Microsomal protein content was determined by the method of Lowry et al. [34].

Ethoxyresorufin 7-deethylase activity (EROD) was measured fluorometrically in rat liver microsomes, using 7-ethoxyresorufin as substrate in order to assess cytochrome CYP1A1-dependent activity [35].

Methoxyresorufin 7-demethylase activity (MROD), which is mainly catalyzed by cytochrome CYP1A2, was determined fluorometrically in the rat liver microsomes according to Burke and Mayer [35].

Total P450 content was determined from CO-differential spectra of dithionite-reduced samples following the method, which was described by Omura and Sato [36]

### Primary hepatocyte cultures

Primary hepatocytes were isolated from rats and used in cultures according to the method of Klaunig et al. [37], [22]. In brief, as previously described by Daskalopoulos et al. [22], primary hepatocytes were isolated from rats weighing 250–300 g using a two-step collagenase perfusion method. They were suspended in William's Medium E (Gibco) containing 1% L-glutamine (PAA) and 1% penicillin/streptomycin. The cells were counted in a Neubauer cell chamber and plated at a density of 1 x 10^5^ cells per well, in 3.8 square centimeter diameter collagen type I coated dish (BIOCOAT, Cell Environment, Becton Dickinson Labware, UK). The viability of the isolated hepatocytes was checked with trypan blue dye 0.4% exclusion and only cells with viability higher than 85% just before plating were used. Hepatocytes were cultured at 37°C for 24 hours under an atmosphere of humidified 5% CO_2,_ in order to allow them to adhere to the wells. Time and dose response experiments started 24 hours later. Primary hepatocyte cultures were treated either with SULP (10 or 25μM) or insulin (1μM) [8], in combination with wortmannin (1μM), an inhibitor of the PI3K/AKT signalling pathway. Wortmannin was added 30 min prior to insulin. Primary hepatocytes were also cultured in the presence of B[a]P at doses raging between 1 and 10μM. Either SULP or insulin were also added in the cell cultures 30 min prior to B[a]P. Time response experiments were conducted with SULP treatment of primary hepatocytes ranging between 1 and 24 hours. These *in vitro* experiments employing primary hepatocytes were approved by the Institutional Animal Care and Use Committee of the Medical School of the University of Ioannina.

### Western blot analysis

Immunoblot analysis of the cytochrome CYPs, STAT5b and FOXO1 apoprotein levels was carried out using microsomes (CYPs) and nuclear extracts or cytosol of liver samples, respectively. For the preparation of the nuclear extracts and cytosol the NE-PER nuclear extraction kit (Pierce, Rockford, IL) was used. The content of the phosphorylated AKT and p70S6K was determined by western blot in total cellular proteins, extracted from the liver using RIPA buffer supplemented with protease inhibitors, PMSF (10μM), BGP (50μM) and NaF (50μM). Protein concentrations were determined by the BCA protein assay (Pierce, Rockford, IL). Proteins were subjected to SDS-PAGE gel electrophoresis and immunoblotting using the following antibodies: rat polyclonal CYP1A1 and CYP1A2 IgGs (they were kindly donated by Dr Ronald Wolf and Colin J. Henderson, London, UK), rat monoclonal total STAT5a/b IgG (Santa Cruz Biotechnology) and rabbit monoclonal p-STAT5b IgG (Tyr 694, Cell Signalling Technology), rabbit polyclonal p-p70S6K IgG (Thr 389), rabbit polyclonal total p70S6K IgG (Cell Signalling Technology), rabbit polyclonal p-FOXO1 (Ser 256) and total FOXO1 IgGs (Santa Cruz Biotechnology), as well as rabbit polyclonal p-AKT (Ser 473), total AKT IgGs (Santa Cruz Biotechnology) and rabbit monoclonal p-mTOR IgG (Ser2448, Cell Signalling) were also used. Secondary antibodies, conjugated with horseradish peroxidase (Santa Cruz Biotechnology) were used and the proteins were detected using a chemiluminescence detection kit (ECL, Amersham, GE Healtcare). Immunoblotting with GAPDH (Santa Cruz Biotechnology) and anti-mouse IgG horseradish peroxidase conjugated secondary antibody, were used as loading control. The membranes were developed by chemiluminescence using the Phototope-HRP Detection Kit for Western blotting (Biolabs INC, New England) and exposed to film.

### Quantitative real-time PCR

The TRIzol reagent (Invitrogen) following the manufacturer’s protocol was used for the isolation of total RNA from liver tissue and primary hepatocytes. A spectrophotometric method was used for the determination of total RNA concentration in each sample. Quantitative real-time reverse transcriptase PCR (qPCR) was performed with cDNA generated from 1 μg total RNA with a SuperScript II reverse transcriptase kit (Invitrogen). The sequences of the forward and reverse gene-specific primers, which were used are shown in S1 Table. For the real-time PCR reactions the SYBR Green PCR master mix was used (Applied Biosystems, Warrington, UK). These reactions were carried out using the Thermal Cycler Real-Time Detection System C1000 (BioRad, Italy). Relative mRNA expression levels were normalized to β-actin (QuantiTect Primer Assay, Qiagen) and values were quantified using the comparative threshold cycle method.

### Hormonal determinations

The GH serum levels were assessed using the rat growth hormone RIA kit (Millipore, MA, USA). The detection limit was 0.5 ng/ml and the intra-assay coefficient of variation was 10%. Prolactin (PRL) serum concentration was determined using the rat prolactin RIA kit (MP Biomedicals Europe, France) and the detection limit was 0.5 ng/ml. Serum thyroid hormone concentrations were determined using the Dynatest T3, Dynatest T4 and Dynatest TSH kits (Brahms, Germany). The normal ranges were 80–200 ng/dl (Dynatest T3), 4.5–12 μg/dl (Dynatest T4) and 0.4–4 mg/ml (Dynatest TSH), respectively. The insulin levels were measured using an EIA kit (Mercodia Rat ELISA kit for insulin, Uppsala, Sweden). The detection limit was 3.3ng/ml and the intra-assay coefficient of variation was 3.1%. The blood glucose levels were measured with a commercially available kit (Merck, Germany) using the technique of glucose oxidase (Trinder, 1969).

### Statistical analysis

The data were expressed as means±SE and were analysed using one-way analysis of variance (ANOVA) followed by multiple comparisons with Bonferoni’s and Tuckey’s list honest significant difference methods. The significance level for all analyses was set at probability of less than or equal to 0.05.

## Results

**Fig 1:**
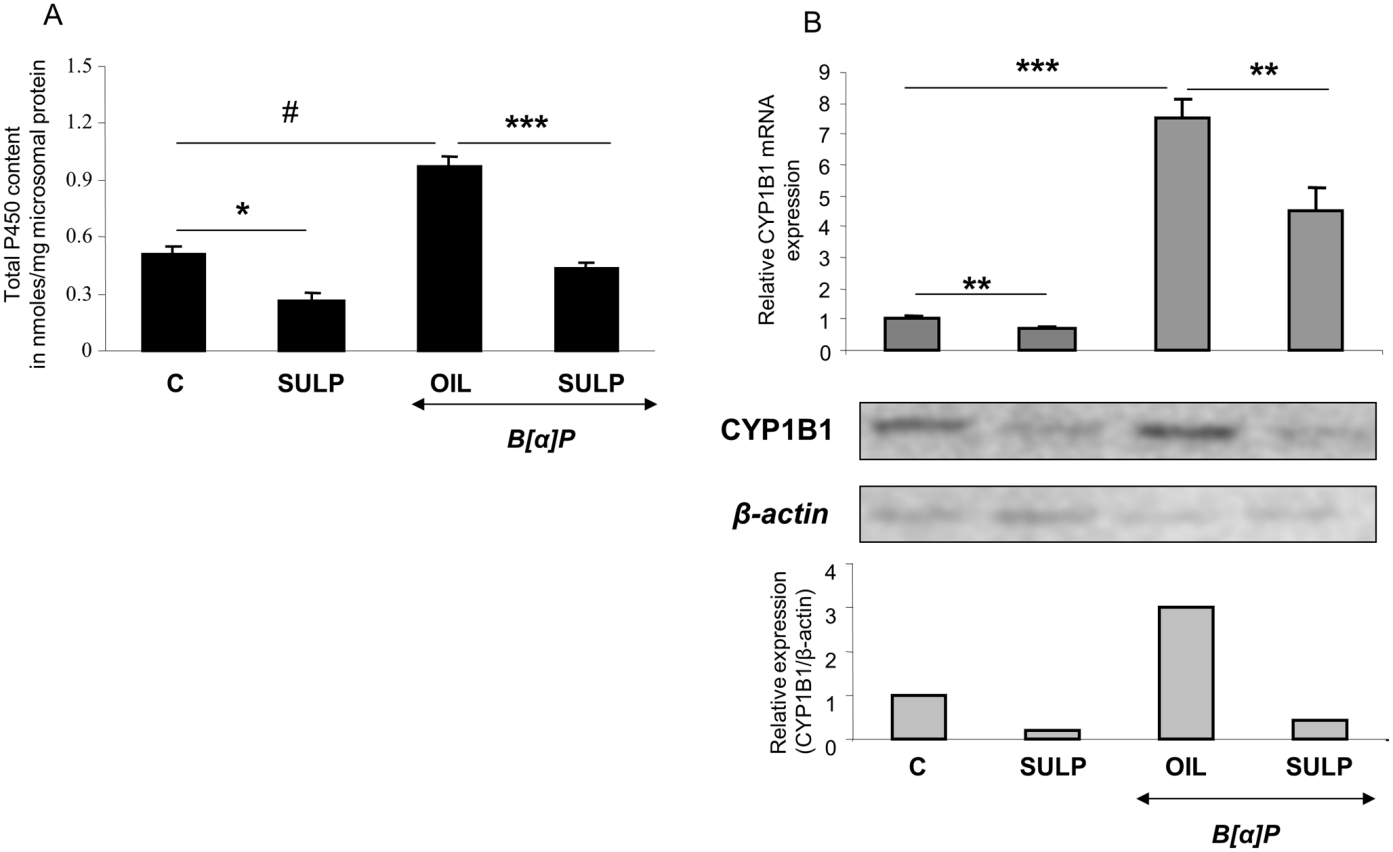
D_2_-dopaminergic receptor-mediated effect on hepatic total P450 content and CYP1B1. (A) Assessment of sulpiride effect on total P450 content in the liver of rats treated with either normal saline or benzo[a]pyrene. (B) Assessment of sulpiride effect on hepatic CYP1B1 mRNA expression following treatment with either normal saline or benzo[a]pyrene. Bonferroni’s correction and Tukey post-hoc tests took place in the comparisons of the data presented here. (C vs SULP, C vs B[a]P and B[a]P vs (B[a]P+SULP)). C: controls treated with normal saline; SULP: sulpiride (dopamine D_2_-antagonist); B[a]P: benzo[a]pyrene; OIL: olive oil; *P<0.05, **P<0.01, #P<0.005, ***P<0.001.

SULP, a dopamine D_2_- receptor antagonist, reduced total P450 content in the rat liver and prevented the B[a]P-induced increase in it (Fig 1A). These effects suggest that one or more hepatic Cytochrome P450 isoforms are down-regulated following blockade of the dopamine D_2_-receptor-linked signalling pathways.

### Blockade of D_2_-dopaminergic receptors down-regulates CYP1A1/2 and CYP1B1

**Fig 2:**
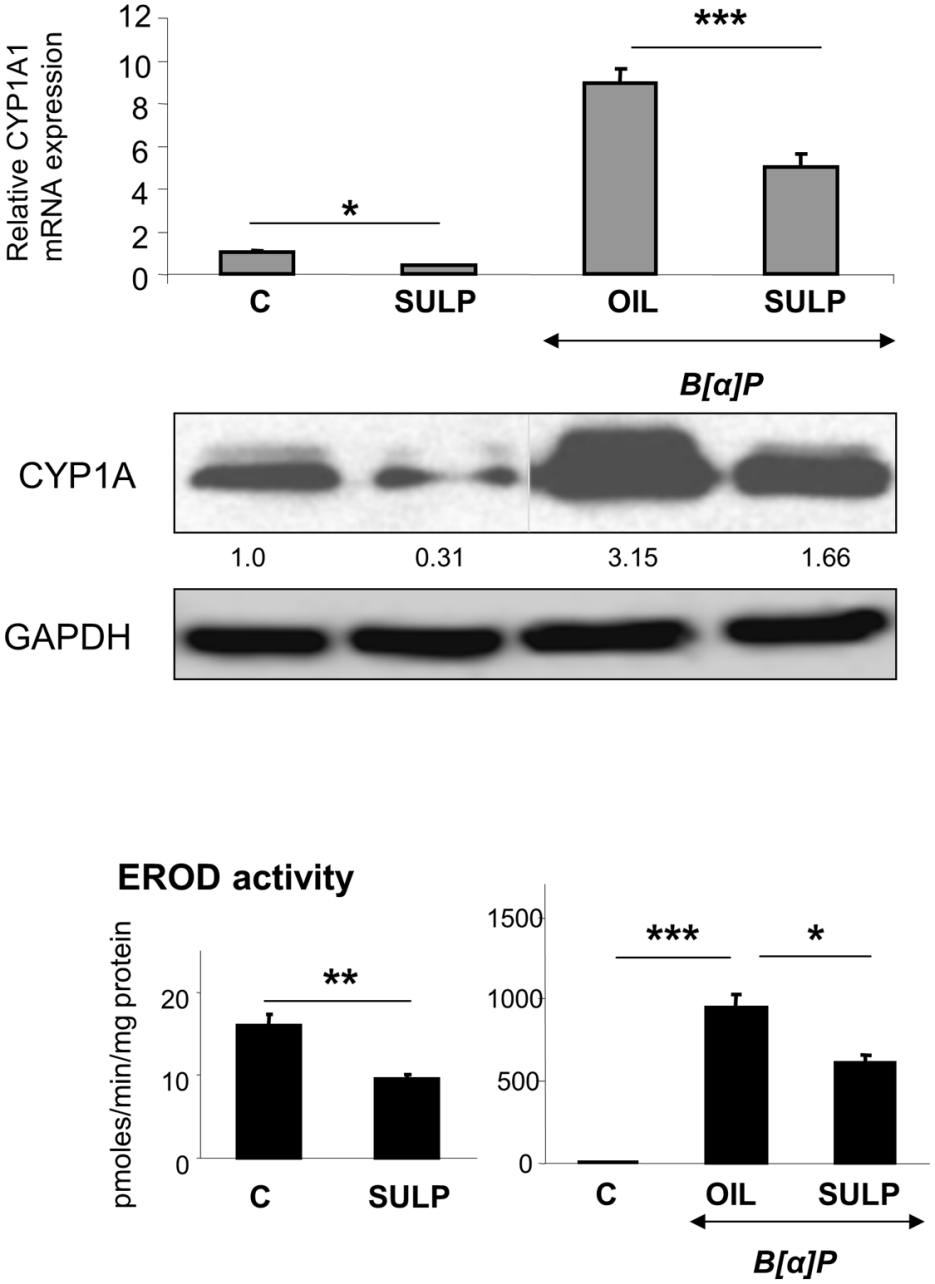
D_2_-dopaminergic receptor-mediated regulation of hepatic CYP1A1. Assessments of the effects induced by the selective dopamine D_2_-antagonist sulpiride (SULP) on *CYP1A1* relative mRNA expression by using quantitative PCR assays, on CYP1A1 apoprotein levels by using Western blotting, and on CYP1A1-catalyzed EROD activity by using a fluorometric assay. Bonferroni’s correction and Tukey post-hoc tests took place in the comparisons of the data presented here (C vs SULP, C vs B[a]P and B[a]P vs (B[a]P+SULP)). Numbers in the western blot captures, correspond to the lower lanes and indicate the relative CYP1A apoprotein expression following treatment compared to the control expression level that was set at 1. The antibody used against CYP1A1 is not highly specific and recognizes CYP1A2 as well. C: controls treated with normal saline; SULP: sulpiride (dopamine D_2_-antagonist); B[a]P: benzo[a]pyrene; OIL: olive oil; *P<0.05, **P<0.01, ***P<0.001.

**Fig 3:**
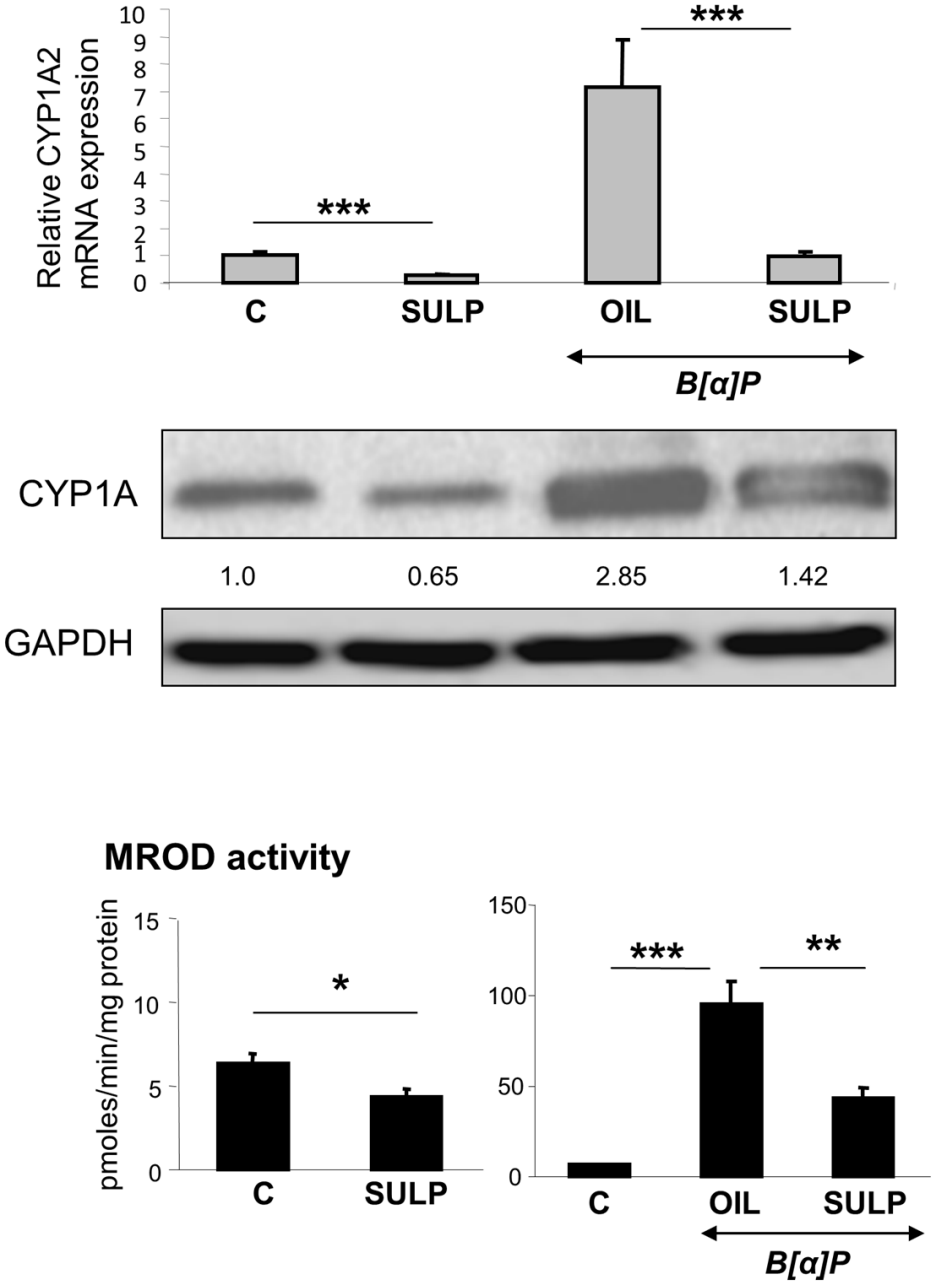
D_2_-dopaminergic receptor-mediated regulation of hepatic CYP1A2. Assessments of the effects induced by the selective dopamine D_2_-antagonist sulpiride (SULP) on *CYP1A2* relative mRNA expression by using quantitative PCR assays, on CYP1A2 apoprotein levels by using Western blotting, and on CYP1A2-catalyzed MROD activity by using a fluorometric assay. Bonferroni’s correction and Tukey post-hoc tests took place in the comparisons of the data presented here (C vs SULP, C vs B[a]P and B[a]P vs (B[a]P+SULP)). Numbers in the western blot captures indicate the relative CYP1A apoprotein expression following treatment compared to the control expression level that was set at 1. C: controls treated with normal saline; SULP: sulpiride (dopamine D_2_-antagonist); B[a]P: benzo[a]pyrene; OIL: olive oil; *P<0.05, **P<0.01, ***P<0.001.

Treatment of rats with the selective D_2_-antagonist SULP, markedly repressed constitutive *CYP1A1* and *CYP1A2* expression in the liver (Figs 2 and 3). This effect was evident at mRNA, apoprotein and enzyme activity (EROD and MROD) levels and was almost identical for both cytochromes. Furthermore, SULP markedly restricted the hepatic B[a]P-induced EROD and MROD activities (Figs 2 and 3). D_2_-receptor blockade also repressed the constitutive and B[a]P-induced *CYP1B1* mRNA and protein expression in the rat liver (Fig 1B). These findings suggest that the D_2_-receptor-related signalling pathways potentially interfere with the mechanism of AhR activation.

**Fig 4:**
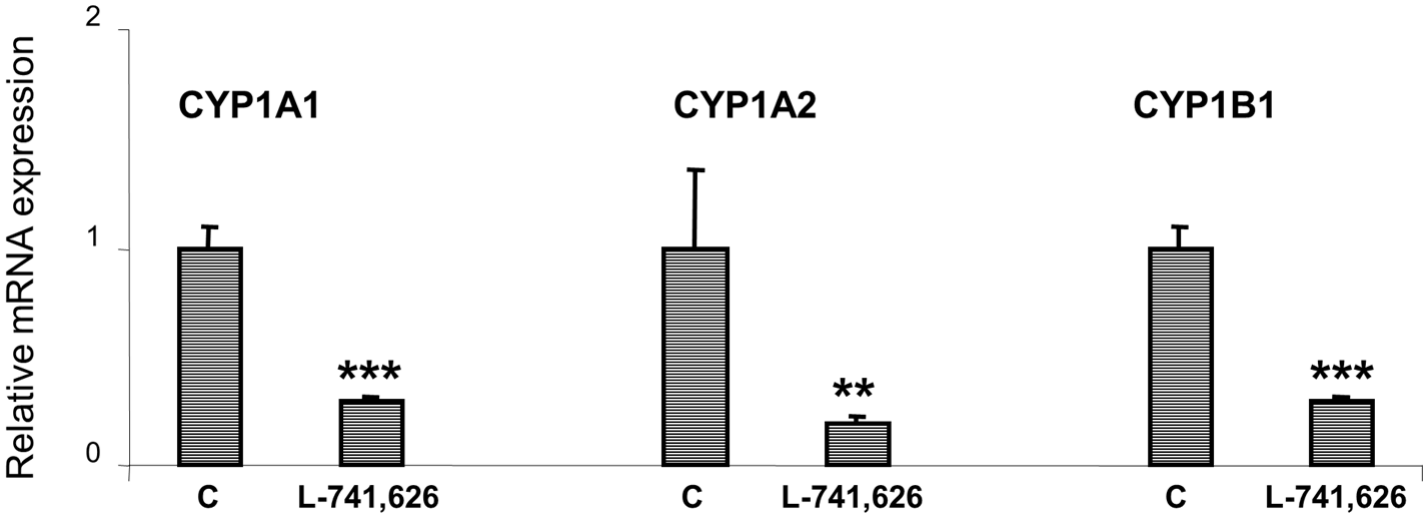
D_2_-dopaminergic receptor-mediated regulation of hepatic CYP1A1/2 and CYP1B1. Assessments of the effects induced by the selective dopamine D_2_-antagonist L-741,626 on *CYP1A1*, *CYP1A2* and *CYP1B1* relative mRNA expression by using quantitative PCR assays. Bonferroni’s test took place in the comparisons of the data presented here (C vs L-741,626). C: controls treated with normal saline; L-741,626: highly selective dopamine D_2_-antagonist; ***P<0.001.

In order to ensure that the effect of SULP on CYP1A1, CYP1A2 and CYP1B1 regulation is mediated by D_2_-dopaminergic receptors, animals were treated with the highly selective dopamine D_2_-antagonist, L-741,626. The data confirmed that blockade of D_2_-dopaminergic receptors results in significant repression of *CYP1A1*, *CYP1A2* and *CYP1B1* mRNA expression (P<0.001, Fig 4), indicating that the down-regulating effect of SULP is, indeed, mediated by inhibition of dopamine D_2_-receptors.

### *In vivo* inhibition of dopamine D_2_-receptors down-regulates the hepatic AhR-dependent CYP regulation system

**Fig 5:**
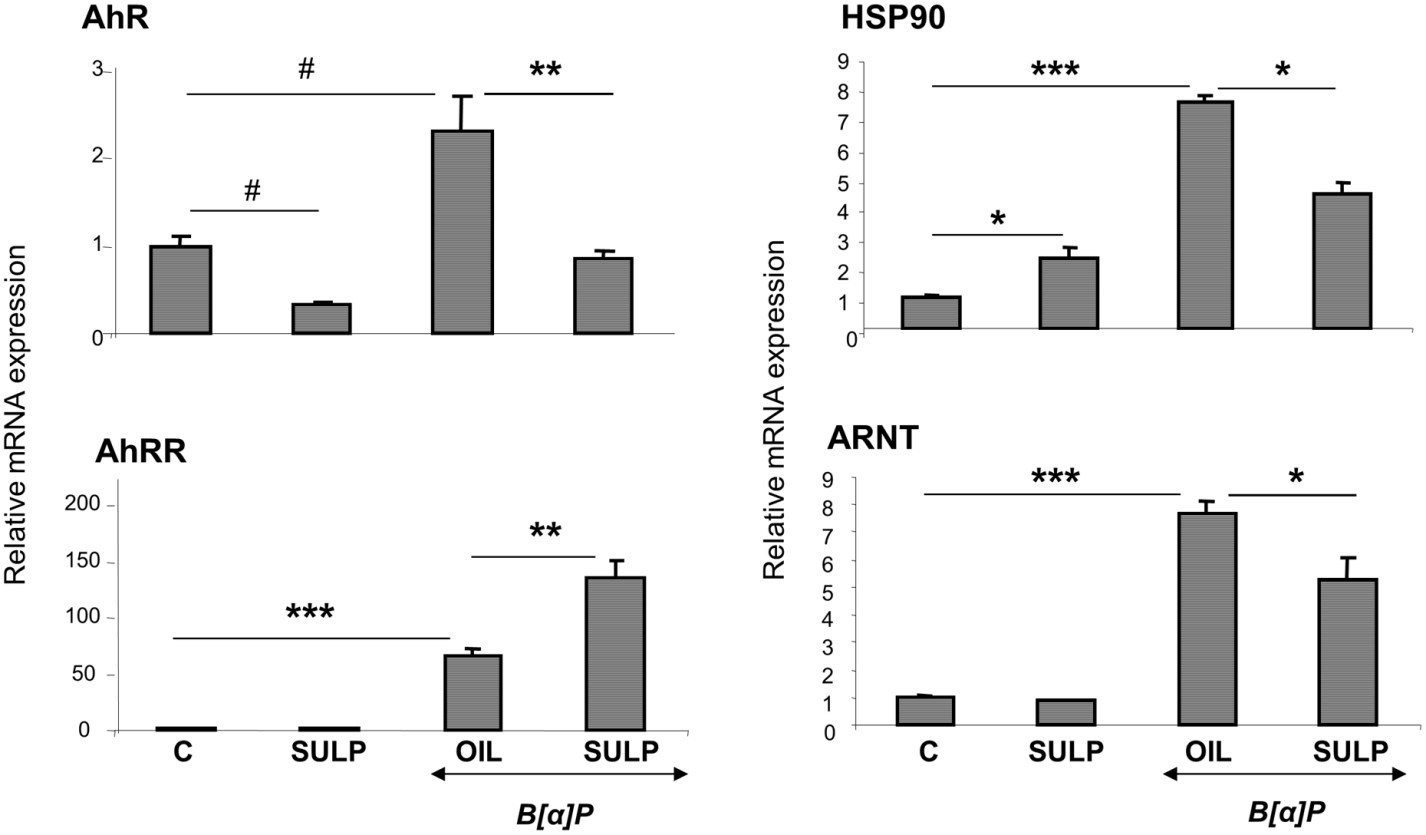
D_2_-dopaminergic receptor-mediated regulation of critical hepatocyte factors that regulate CYP1A1, CYP1A2 and CYP1B1. Assessments of the effects induced by the selective dopamine D_2_-antagonist sulpiride on aryl hydrocarbon receptor *[1]*, aryl hydrocarbon receptor repressor *(AhRR)*, aryl hydrocarbon receptor nuclear translocator *(ARNT*) and heat shock protein 90 *(HSP90)* relative mRNA expression by using quantitative PCR assays. Bonferroni’s correction and Tukey post-hoc tests took place in the comparisons of the data presented here. In particular, comparisons of data (Relative ARNT and AhRR mRNA levels) between the group of controls (C) and SULP took place using the Bonferroni’s and Tukey tests and no difference was found. (C vs SULP, C vs B[a]P and B[a]P vs (B[a]P+SULP)). C: controls treated with normal saline; SULP: sulpiride (dopamine D_2_-antagonist); B[a]P: benzo[a]pyrene; OIL: olive oil; *P<0.05, **P<0.01, #P<0.005, ***P<0.001.

D_2_-receptor blockade with SULP resulted in repression of *AhR*, heat shock protein 90 (*HSP90*) and aryl hydrocarbon receptor nuclear translocator (*ARNT*) in the liver of rats exposed to B[a]P (Fig 5). In contrast, SULP further increased the B[a]P-induced AhR repressor *(AhRR)* mRNA expression (Fig 5). Interestingly, SULP did not affect *ARNT* and *AhRR* constitutive mRNA expression, whereas up-regulated *HSP90* basal expression (Fig 5).

### Role of the insulin/PI3K/AKT/FOXO1 pathway, in the D_2-_receptor-mediated down- regulation of CYP1A and CYP1B

**Fig 6:**
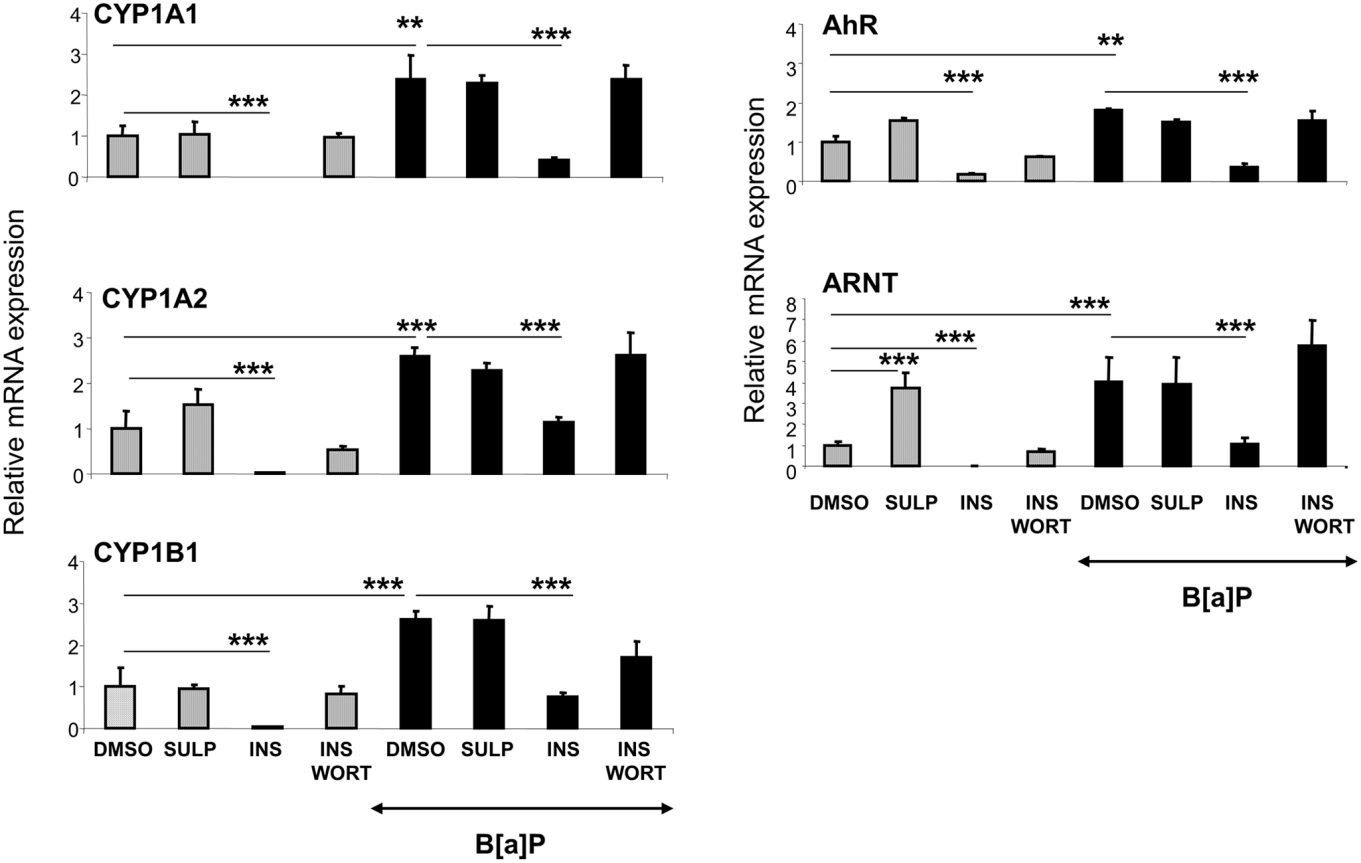
*In vitro* assessment of the role of dopamine D_2_-receptor-mediated regulation of hepatic CYP1A1, CYP1A2 and CYP1B1. Assessments of SULP effects on *CYP1A1*, *CYP1A2* and *CYP1B1* relative mRNA expression in primary hepatocytes by using quantitative PCR assays. The role of insulin in the regulation of the above mentioned CYPs was also assessed in primary hepatocyte cultures treated either with insulin (1μM, 24hr) alone or in combination with the inhibitor of the PI3K signalling pathway, wortmannin (1μM, 24hr) [76]. Controls were treated with dimethylsulfoxide (DMSO); SULP: sulpiride (selective dopamine D_2_-antagonist); INS: insulin; WORT: wortmannin; B[a]P: benzo[a]pyrene; ***P<0.001. Comparisons of the relative *CYP1A1*, *CYP1A2*, *CYP1B1*, *AhR* and *ARNT* mRNA expression between DMSO- and INS-, as well as between DMSO- and (INS+WORT)-treated hepatocytes were done using the Bonferroni’s test and were performed in both, constitutive and B[a]P-induced states. No differences were found between DMSO- and (INS+WORT)-treated hepatocytes, indicating that wortmannin has completely blocked the repressive effect of insulin on both, constitutive and B[a]P-induced mRNA expression of the above mentioned *genes*.

In order to elucidate the mechanism underlying the down-regulation of CYP1 enzymes following inhibition of D_2_-receptors, *in vitro* experiments were employed using primary hepatocyte cultures. Time- and dose-response experiments were conducted for the determination of the optimal conditions for the treatment of primary hepatocytes with SULP (S1 and S2 Figs). It was found that treatment of hepatocytes with SULP did not alter the constitutive and B[a]P-induced expression of *CYP1A1/2* and *CYP1B1* (Fig 6). The findings of this *in vitro* study, where SULP affected directly the hepatocytes, indicated that the down-regulation of the CYP1 *genes* observed following the *in vivo* SULP administration appears to involve primarily extrahepatic D_2_-receptor linked pathways.

**Fig 7:**
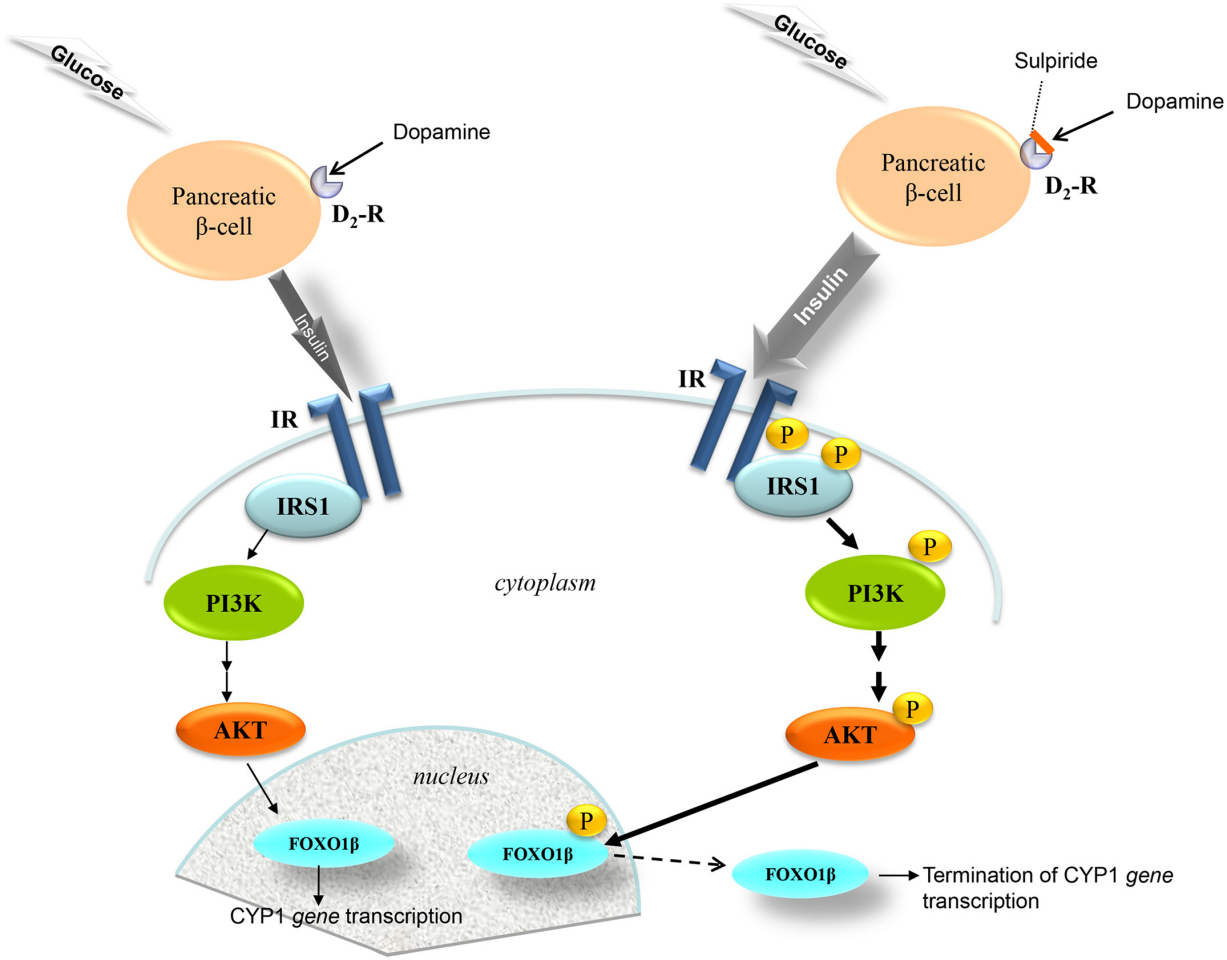
The dopamine D_2_-receptor-mediated control of the insulin/PI3K/AKT/FOXO1 signalling pathway activation. Dopamine stimulates D_2_-ARs on pancreatic β-cells and restricts the release of insulin in response to increased plasma glucose levels [61]. In contrast, blockade of D_2_-dopaminergic receptors by sulpiride, increases insulin release [22], which in turn, stimulates insulin receptors (IR) in hepatocyte plasma membranes, an effect resulting in the phosphorylation of the Insulin Receptor Substrate (IRS) at different docking sites, where the phosphatidylinositol 3-kinase (PI3K) binds. Activated PI3K converts phosphatidylinositol biphosphate to phosphatidylinositol triphosphate, which subsequently activates protein kinase B (AKT). Upon activation AKT phosphorylates the transcription factor forkhead box O1 (FOXO1), which then translocates into the cytoplasm thus terminating *CYP1A1*, *CYP1A2* and *CYP1B1* gene transcription [22,75].

**Fig 8:**
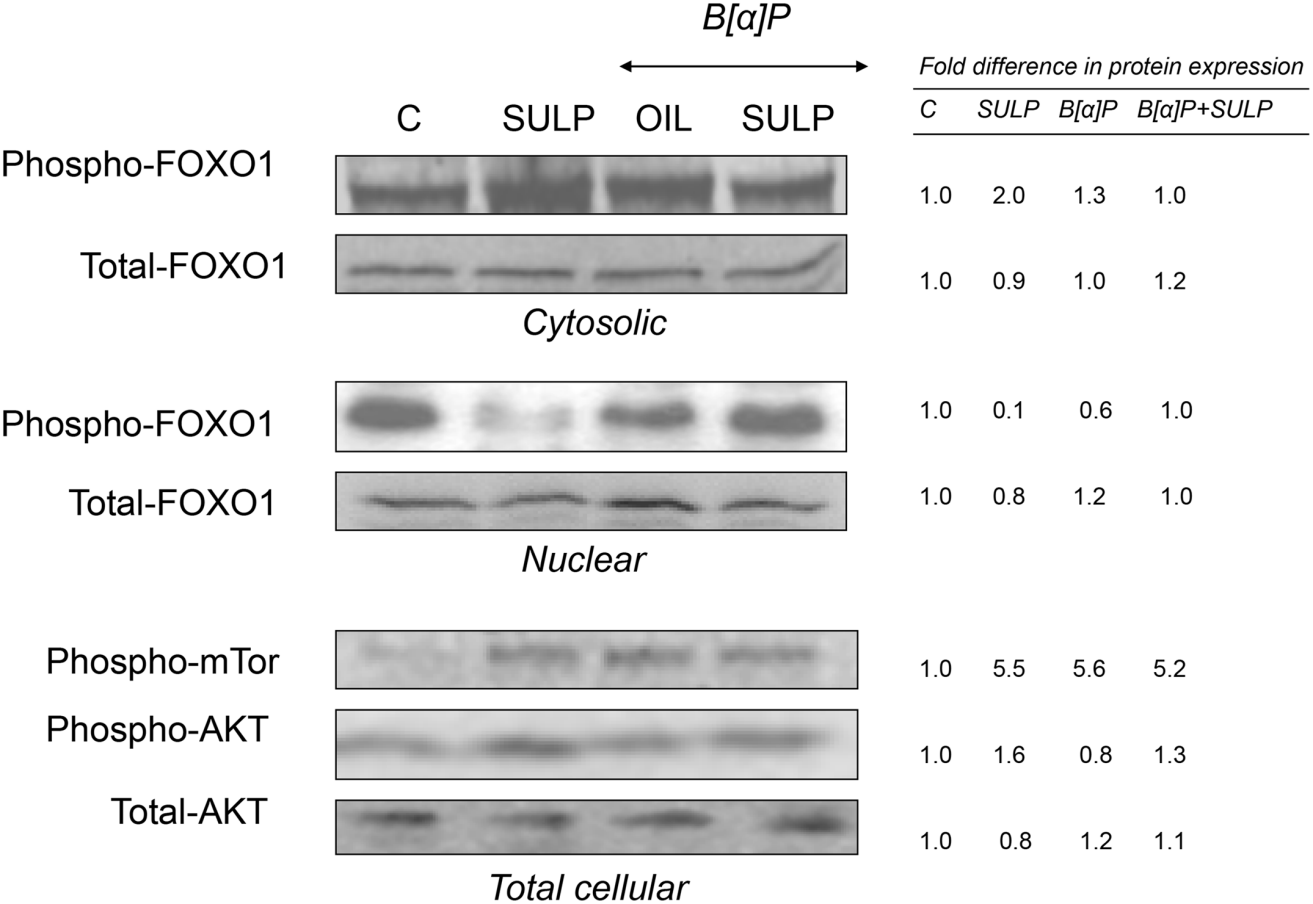
*In vivo* assessment of the effect of D_2_-receptor blockade on the insulin/PI3K/AKT/FOXO1 and mTOR signalling pathway. Western blotting showing the SULP-mediated AKT phosphorylation that activated FOXO1 in the nucleus, which in turn translocated into the cytoplasm (reduced FOXO1β phosphorylation in the nucleus and increased FOXO1β phosphorylation in the cytoplasm). Numbers in the western blot captures indicate the relative FOXO1β, AKT and mTOR phosphorylation level following treatment compared to the control level that was set at 1. C: controls treated with normal saline; SULP: sulpiride (selective dopamine D_2_-antagonist); B[a]P: benzo[a]pyrene; OIL: olive oil.

The findings coming from the *in vivo* study indicate that the pancreatic β-cell D_2_-receptor/insulin-related pathway potentially has a central role in the SULP-mediated repressive effect on the constitutive and B[a]P-induced *CYP1A1*, *CYP1A2* and *CYP1B1* expression. It is well documented that SULP by blockade of pancreatic islet β-cell D_2_-receptors increases insulin release (Fig 7), an effect that was confirmed by the present study (Table 1; [22]). It was also found that treatment of rats with SULP activated the insulin/PI3K/AKT/FOXO1 signalling pathway in the liver (Fig 7). Specifically, SULP increased AKT phosphorylation that subsequently activated FOXO1β in the nucleus, thus leading to its translocation into the cytoplasm, and termination of CYP1 *gene* transcription at a constitutive level (Fig 8). This hypothesis is also supported by the finding that treatment of primary hepatocytes with insulin strongly repressed *CYP1A1*, *CYP1A2*, *CYP1B1*, *AhR* and *ARNT* mRNA expression, effects that were completely prevented by wortmannin, a PI3K inhibitor, able to block the insulin/PI3K/AKT/FOXO1 signalling pathway (Fig 6), [38]. SULP also activated AKT and in turn, increased FOXO1β phosphorylation in the nucleus of hepatocytes of B[a]P-exposed rats, indicating an activation of the PI3K/AKT signalling pathway (Fig 8). *In vitro* treatment of primary hepatocytes with insulin (it is released *in vivo* following SULP treatment, [22]), resulted in a strong restriction of CYP1 inducibility by B[a]P, an effect that was prevented by wortmannin, a PI3K inhibitor (Fig 6).

**Table 1:** Sulpiride-induced effect on rat hormonal state.

Treatment	T3	T4	TSH	PRL	GH	CORT	Insulin
Control	130.7±6.8	2.9±0.1	2.1±0.1	38.4±6.4	129.8±3.5	193.1±14.4	0.44±0.02
SULP	78.54±5.6***	1.6±0.2***	1.9±0.1	194.9±13.0***	41.1±12.3***	85.1±11.0**	1.4±0.2***
B[a]P	93.9±5.5***	2.5±0.1*	2.1±0.01	28.9±4.4	110.8±9.1	196.8±19.4	0.35±0.03
B[a]P + SULP	65.0±3.5**	1.3±0.1***	2.1±0.01	247.7±12.8***	59.7±0.3*	102.3±2.0*	1.5±0.2***

C; controls treated with normal saline; SULP: sulpiride (dopamine D_2_-antagonist); B[a]P: benzo[a]pyrene; OIL: olive oil; T3: triiodothyronin expressed in ng/dl; T4: thyroxin expressed in μg/dl; TSH: thyroid-stimulating hormone expressed in ng/ml; GH: growth hormone expressed in ng/ml; PRL: prolactin expressed in ng/ml; Corticosterone expressed in mg/ml; Insulin expressed in pg/ml. Values are expressed as mean ± SE (n = 10). The asterisks indicate the significance of the differences between SULP-treated rats and controls, and between B[a]P-exposed rats with and without concomitant treatment with SULP

*P<0.05

**P<0.005

***P<0.001

Taken together these data indicate that the mechanism of SULP-mediated down-regulation of *CYP1A* and *CYP1B* constitutive expression potentially involves activation of the hepatic insulin/PI3K/AKT/FOXO1β signalling pathway. This activation, however, may not apply for the SULP-mediated restriction of the CYP1 inducibility by B[a]P. It is possible that factors, other than FOXO1β, downstream to the PI3K/AKT signalling pathway are involved and hold predominant roles in the SULP-mediated repression of CYP1 inducibility.

Based on the fact that there is a cross-talk between mTOR/HIF1a and the AhR-linked signalling pathway [39–41], the effect of SULP on mTOR phosphorylation and *HIF1a* expression was investigated. As mentioned above, D_2_-receptor blockade with SULP activated the PI3K/AKT signalling pathway in non exposed to B[a]P livers (Fig 8), an activation also seen at the level of mTOR (a down-stream element in this pathway). It should be noted though, that SULP did not alter the level of mTOR phosphorylation in the B[a]P-exposed livers (Fig 8). Furthermore, this D_2_-receptor antagonist did not alter both, constitutive and B[a]P-induced *HIF1a* mRNA expression in the rat liver (S3 Fig). Apparently, this cross-talk is not critically involved in the SULP-mediated down-regulation of CYP1 inducibility by B[a]P.

### Dopamine D_2_-receptor blockade inactivates the GH/STAT5b signalling pathway

**Fig 9:**
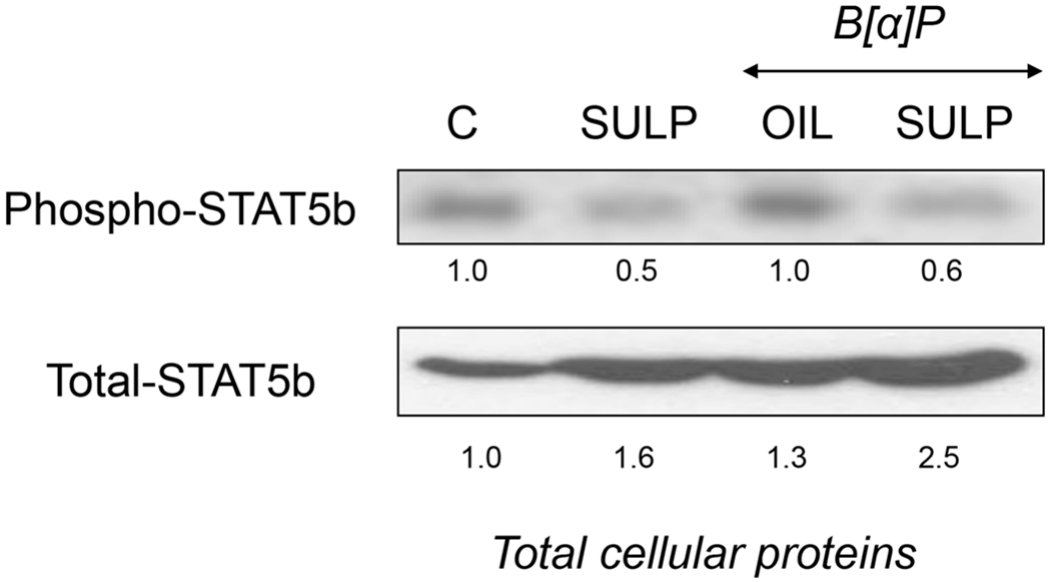
*In vivo* assessment of the effect of D_2_-receptor blockade on the activation of GH/STAT5b signalling pathway. Western blotting showing the SULP-mediated suppression of STAT5b phosphorylation. Numbers in the western blot captures indicate the STAT5b phoshorylation level following treatment compared to the control level that was set at 1. Lanes C: control; SULP: sulpiride (selective dopamine D_2_-antagonist); B[a]P: benzo[a]pyrene; OIL: olive oil.

It is well established that dopamine stimulates the secretion of growth hormone (GH) from the anterior-pituitary lobe [42], which has a down- regulating effect on hepatic *CYP1A* expression via the Jak2/STAT5b pathway [43]. In the present experimental setting, SULP reduced serum GH levels (Table 1) and consequently, STA5b phosphorylation both, in B[a]P-exposed and non-exposed rats (Fig 9). Based on these findings a CYP1A and CYP1B up-regulation should be expected. However, the opposite is true indicating that the pancreatic D_2_-receptor-insulin-PI3K/AKT/FOXO1 pathway plays a dominant role overriding that of the GH/STAT5b pathway.

### Effect of dopamine D_2_-receptor blockade on neurochemical and hormone levels

The role of hormones, such as GH, thyroid hormones, PRL and glucocorticoids in the regulation of CYP1A and CYP1B isozymes is well documented [26,44–48]. The secretion of these hormones is under a central noradrenergic and dopaminergic control [42]. Therefore, the levels of norepinephrine (NE) and dopamine (DA) were determined in the hypothalamus, the brain site where the corresponding hormone releasing factors are released from [42].

SULP reduced NE content in the hypothalamus of both, B[a]P-exposed and non-exposed rats (P<0.05 and P<0.01, respectively, Table 2). Hypothalamic DA levels were also reduced by SULP only in the non-exposed to B[a]P rats (P<0.05, Table 2), whereas DA turnover ratio was increased in this group of treatment (P<0.01, Table 2), indicating an increased dopaminergic activity in the hypothalamus.

**Table 2:** Sulpiride-induced effect on hypothalamic catecholamines, norepinephrine (NE) and dopamine (DA).

	Control	SULP	B[a]P	B[a]P+ SULP
NA	182.0±19.4	76.8±3.3**	281.8±16.6	200.0±11.1*
DA	18.9±2.3	8.1±1.5*	19.4±0.7	16.1±1.1
DOPAC	13.3±1.0	17.4±0.1	11.1±0.7	13.8±1.1
HVA	3.3±0.5	3.1±0.2	2.0±0.1	3.5±0.4
Turnover DA	0.9±0.13	2.6±0.4**	0.7±0.1	1.1±0.1

The evaluation of the effect of sulpiride (SULP), a dopamine D_2_-antagonist, on hypothalamic dopaminergic activity was based on the alterations observed at DA turnover ratio (HVA+DOPAC)/DA. DA, NE, DOPAC and HVA levels were expressed in nmoles/g tissue. HVA: homovanilic acid; DOPAC: dihydroxyphenylacetic acid. Values are expressed as mean ± SE (n = 10). The asterisks indicate the significance of the differences between SULP-treated rats and controls, and between benzo[a]pyrene (B[a]P)-exposed rats with and without concomitant treatment with SULP;

*P< 0.05

**P< 0.01

***P< 0.001

SULP reduced serum T3, T4, GH and corticosterone levels both, in B[a]P-exposed and non-exposed rats, but the drug increased PRL and insulin concentration in these rats (Table 1). Interestingly, B[a]P suppressed serum T3 and T4 levels (Table 1).

## Discussion

Previous studies have shown that central and peripheral catecholaminergic systems play a critical role in the regulation of several CYPs that are involved in the metabolism of the majority of prescribed drugs and toxicants. In particular, noradrenergic systems hold prevalent roles in CYP regulation [13,18,20–22,26,49]. Accumulating evidence indicates that dopaminergic systems and mainly those related to dopamine D_2_-receptors, are also involved [22,23]. Specifically, inhibition of D_2_-dopaminergic receptors with either sulpiride or L,741,626 led to a robust down-regulation of CYP2C, CYP2D, CYP2E1, and CYP3A in the rat liver [22,23]. The present study has focused on the role of dopamine D_2_-receptor-linked pathways in the regulation of constitutive and B[a]P-induced *CYP1A1/2* and *CYP1B1* expression. It should be noted that dopamine D_2_-receptors are targets of drugs used in the treatment of various neurodegenerative and psychopathological disorders, such as the Parkinson’s disease, depression and psychosis [50–52]. The drugs prescribed in these diseases are either D_2_-agonists or antagonists that can influence the functional efficiency of the dopaminergic system by mimicking, blocking, or modifying the sensitivity of D_2_-receptors to dopamine.

The present findings indicated that blockade of dopamine D_2_-receptors markedly repressed both, the constitutive and B[a]P-induced expression of *CYP1A1/2* and *CYP1B1*. It is well documented that AhR, a member of the basic helix-loop-helix/PER-ARNT-SIM family of DNA-binding proteins, is a determinant transcription factor that regulates both, constitutive and B[a]P-induced *CYP1A1/2* and *CYP1B1* expression[53]. Transcriptional activation of these CYP isozymes requires firstly binding of the ligand, such as B[a]P, to AhR, which is associated with HSP90 in the cytoplasm. Upon ligand binding the complex translocates into the nucleus where HSP90 dissociates. The ligand-AhR complex then forms a heterodimer with the ARNT [54] and interacts with the xenobiotic responsive elements at the promoters of the CYP1A1/2 and CYP1B1 *genes*, thus inducing their transcription [54,55]. Therefore, the findings of this study indicate that the reduction of CYP1A1/2 and CYP1B1 inducibility by B[a]P that was detected following D_2_-receptor blockade with SULP, and that of AhR, HSP90 and ARNT, indicates a mechanism that profoundly includes impaired transcription of the AhR responsive genes [56]. In support of this hypothesis is the fact that SULP further increased the B[a]P-induced *AhRR* expression. It is well established that contrary to what applies for the induced CYP1 *gene* expression, the induced levels of AhRR inhibit the AhR function by competing with it in forming a heterodimer with ARNT thus compromising its XRE binding activity [57].

The fact that SULP further enhanced the B[a]P-induced *AhRR* expression, instead of suppressing it, as in the case of other AhR target genes, confirms previous observations that the outcome of AhR-AhRR interactions is more complex than suggested by a simple AhR-induced, AhRR-mediated feedback model, which is based on the ability of AhR to transactivate the *AhRR* gene, and the ability of AhRR to repress AhR activity, at least as defined by *CYP1* induction. In particular, it has been reported that the ligand-activated AhR may not transactivate *AhRR* in all tissues and under some circumstances, may actually repress *AhRR* transcription, thereby maximizing AhR activity [58].

It should be noted that AhRR, HSP90 and ARNT do not hold essential roles in the SULP-mediated repression of *CYP1A* and *CYP1B* constitutive expression, because the drug did not affect basal *ARNT* and *AhRR* expressions, whereas increased that of *HSP90*. Profoundly, SULP along with *AhR*, repressed some of the numerous ancillary factors that are involved in the activation of the AhR transcriptional complex that regulates constitutive *CYP1* expression [59]. Finally, it appears that while AhR regulates both, the constitutive and the induced *CYP1* expression, the mechanism mediating the SULP repressive effect on each expression state is not identical. There may be differences at the level of AhR interaction with other transcription factors, or potentially at some upstream signalling pathways, or at the AhR affinity level in binding with the respective gene promoters.

The role of dopamine D_2_-receptor-linked pathways in the down-regulation of hepatic *CYP1A1/2* and *CYP1B1* was confirmed with another, highly selective D_2_-antagonist, the L-741,626, which also repressed their expression. This D_2_-receptor mediated effect is probably indirect, because treatment of primary hepatocyte cultures with SULP had no effect on the expression of *CYP1A1/2* and *CYP1B1*. It is therefore, hypothesized that the repressive effect on *CYP1A1/2* and *CYP1B1*, which is observed when SULP is administered *in vivo*, is the outcome of the drug’s effects on central and peripheral dopaminergic and hormonal systems that have an impact on hepatic signalling pathways regulating these CYP *genes* [22].

Our results suggest a role for the hepatic insulin/PI3K/AKT/FOXO1 signalling pathway in the SULP-mediated down-regulation of constitutive *CYP1A1/2* and *CYP1B1* expression. It has been previously shown that inhibition of pancreatic D_2_-dopaminergic receptors with SULP triggers insulin secretion from pancreatic islet β-cells. This effect leads to activation of the insulin/PI3K/AKT signalling pathway in the hepatocytes [22] and ultimately, to phosphorylation of the nuclear factor FOXO1β and its subsequent translocation into the cytoplasm and termination of *CYP1A1*, *CYP1A2* and *CYP1B1* transcription. Upstream to the insulin/PI3K/AKT/FOXO1 pathway, dopamine is a significant regulatory factor that restricts the release of insulin in response to increased plasma glucose levels (Fig 7). Dopamine, exerts its negative control in insulin release via stimulation of pancreatic D_2_-receptors expressed in islet β-cell membranes [60]. In contrast, blockade of these receptors with SULP increases insulin release (Fig 7), [23], which in turn, activates the hepatic insulin/PI3K/AKT/FOXO1 signalling pathway, thus exerting a negative regulatory control on several P450s [22,60–64]. This hypothesis is also supported by the findings of an *in vitro* study. Treatment of primary hepatocyte cultures with insulin resulted in a strong down-regulation of CYP1A1, CYP1A2 and CYP1B1 via activation of the PI3K/AKT signalling pathway, because pre-treatment of these cells with wortmannin, a PI3K inhibitor, prevented the repressive effect of insulin on these cytochromes. The PI3K/AKT signalling pathway may also participate at least in part, in the SULP-mediated reduction of CYP1A1/2 and CYP1B1 inducibility by B[a]P. This hypothesis is based on the fact that SULP increased AKT and FOXO1β phosphorylation in the liver of B[a]P-exposed rats, indicating an activation of the insulin/PI3K/AKT signalling pathway. Furthermore, insulin represses CYP1 inducibility by B[a]P in primary heatocytes and wortmannin completely blocked this effect. The *in vivo* and *in vitro* findings indicate that profoundly, some distinct downstream elements in the insulin/PI3K/AKT signalling pathway participate in the SULP-mediated repression of CYP1A1/2 and CYP1B1 inducibility by B[a]P than those regulating the repression of *CYP1* constitutive expression. However, further investigation involving potentially Chip assays is needed in order to completely clarify the involvement of PI3K/AKT signalling pathway in CYP1A and CYP1B regulation by SULP.

Previous studies reported a cross-talk between the AhR and HIF1a signalling pathways [39]. It is well established that activation of the PI3K/AKT/mTOR-related pathway up-regulates HIF1a [39,41], which in turn, inactivates AhR, thus repressing CYP1A inducibility [40]. It should be taken also into account the fact that the impact of this cross-talk depends on ARNT availability, which is an essential element in both, AhR and HIF1a signalling pathways [39] and SULP repressed the B[a]P-induced ARNT expression. The present study also indicated that SULP activated mTOR in the liver of non exposed to B[a]P rats, but had no effect on the B[a]P-exposed livers. In addition, SULP did not affect HIF1a inducibility by B[a]P. Combined these data indicate that the HIF1a/mTOR and AhR cross-talk did not have any critical role in the SULP-mediated suppression of the CYP1A1/2 and CYP1B1 inducibility by B[a]P.

In the D_2_-receptor related regulatory system, the cross-talk between the AhR- and insulin/PI3K/AKT-linked signalling pathways apparently, holds significant roles in CYP1 regulation [65–67]. In light of this interaction, it is suggested that the SULP-induced repression of *CYP1A1/2* and *CYP1B1*, could be attributed, at least in part, to the insulin-induced up-regulation of the carbohydrate-responsive element-binding protein (ChREBP) in the liver. This factor exerts a negative control on ARNT/HIF-1β, an essential element in the AhR regulatory system [65].

Stimulation of GH secretion from the anterior pituitary lobe by dopamine is well documented [42]. STAT5b, is the major GH pulse-activated transcription factor, which is involved in the regulation of several P450s in the liver [43,68]. Therefore, the possible involvement of the GH/STAT5b pathway in the SULP-mediated repression of *CYP1A* and *CYP1B1* was assessed. Treatment with SULP resulted in decreased serum GH levels and inactivation of the GH/STAT5b signalling pathway. Thyroid hormone levels were also decreased by SULP. Based on the fact that both, GH and thyroid hormones, hold a negative control on CYP1A regulation [43,69,70], it is assumed that the SULP-induced repression of *CYP1A* and *CYP1B1* is not mediated by inactivation of the GH/STAT5b- and thyroid hormone-related pathways.

Other regulatory pathways are potentially involved including PRL, as SULP increased serum PRL concentration, which has a down-regulating effect on various P450 isoforms [69,71]. Glucocorticoid-related pathways could be also involved, as SULP reduced serum corticosterone concentration, which is a positive regulator of CYP1A1/2 and CYP1B1 in the rat liver [72].

In conclusion, inhibition of dopamine D_2_-receptors results in significant repression of constitutive and B[a]P-induced *CYP1A1*, *CYP1A2* and *CYP1B1* expression in the rat liver. The activation of the AhR-related regulatory system was reduced following D_2_-inhibition, indicating a cross-talk between the D_2_-receptor- and AhR-regulatory pathways. Previous studies indicated that D_2_-receptor-linked signalling pathways potentially cross-talk also with a broad array of cellular transcription systems that regulate various P450 *genes* encoding isozymes that metabolize a plethora of prescribed drugs and toxicants [22], indicating that the down-regulating effect of D_2_-receptor inhibition is not specific for the AhR-related regulatory system. The down-regulating effect of SULP on constitutive *CYP1A* and *CYP1B* expression appears to be mediated by activation of the insulin/PI3K/AKT pathway, which though has a less significant contribution in the SULP-mediated reduction of CYP1 inducibility by B[a]P. The PRL-activated negative regulatory pathway and inactivation of the glucocorticoid-linked up-regulating pathway may also contribute in the SULP-mediated down-regulation of CYP1A and CYP1B. As dopamine D_2_-receptors serve as targets for various prescribed drugs, patients following a relative treatment may have altered drug toxicity and efficacy outcomes due to reduced enzymatic activity when exposed to substrates of the CYP1A1/2 and the CYP1B1. These findings potentially indicate that the regulatory pathways involving D_2_-receptors could be considered as targets of the pharmaceutical strategy for the protection of individuals heavily exposed to environmental toxicants and pre-carcinogens that are activated upon CYP1A- and CYP1B-catalyzed metabolism. It should be noted though that carcinogenesis is a complex and multi-factorial process that involves various regulatory systems, and not only those related to CYP1A1/2 and CYP1B1 enzymes [73,74]. Therefore, further investigation is needed in order to clarify the outcome of D_2_-receptor inhibition in carcinogenesis, such as assessment of the B[a]P-DNA-adduct formation in the liver and other tissues.

## Supporting Information

S1 Fig Time response variation in CYP1 *gene* expression following sulpiride. Alterations in CYP1A1, CYP1A2 and CYP1B1 relative mRNA expression following sulpiride (SULP, 10μM) exposure of primary hepatocytes for different time periods was assessed. Control cells were treated with DMSO. SULP1: incubation of hepatocytes with sulpiride for 1 hr; SULP6: incubation of hepatocytes with sulpiride for 6 hr; SULP12: incubation of hepatocytes with sulpiride for 12 hr; SULP24: incubation of hepatocytes with sulpiride for 24 hr; ***P<0.001.
(DOC)

S2 Fig Dose response variation in CYP1 *gene* expression following sulpiride. Alterations in CYP1A1, CYP1A2 and CYP1B1 relative mRNA expression following exposure of primary hepatocytes with either 10μM or 25μM sulpiride (SULP) for 24 hr. Control cells were treated with DMSO.
(DOC)

S3 Fig Sulpiride effect on constitutive and B[a]P-induced *HIF1a* mRNA expression in the rat liver. ***P<0.001.
(DOC)

S1 Table Nucleotide sequences of the primers used. These primers were used for the quantitation of the mRNA levels of the *genes* tested using q-PCR.
(DOC)
