# Supplementary figures and images for: Dopamine D2-Receptor Antagonists Down-Regulate CYP1A1/2 and CYP1B1 in the Rat Liver
Source: PLoS One. 2015 Oct 14;10(10):e0128708. doi: 10.1371/journal.pone.0128708 (PMC4605514; doi:10.1371/journal.pone.0128708)

**S1 Figure.** **Time response variation in CYP1 *gene* expression following sulpiride.**


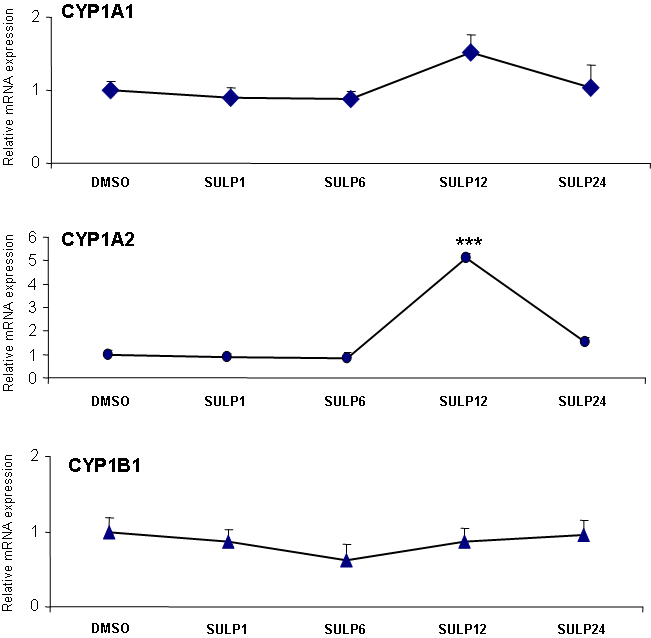

Supplement: S1 Fig — Alterations in CYP1A1, CYP1A2 and CYP1B1 relative mRNA expression following sulpiride (SULP, 10μM) exposure of primary hepatocytes for different time periods was assessed. Control cells were treated with DMSO. SULP1: incubation of hepatocytes with sulpiride for 1 hr; SULP6: incubation of hepatocytes with sulpiride for 6 hr; SULP12: incubation of hepatocytes with sulpiride for 12 hr; SULP24: incubation of hepatocytes with sulpiride for 24 hr; ***P<0.001. (DOC) [file pone.0128708.s001.doc]

**S2 Figure.** **Dose response variation in CYP1 *gene* expression following sulpiride.**


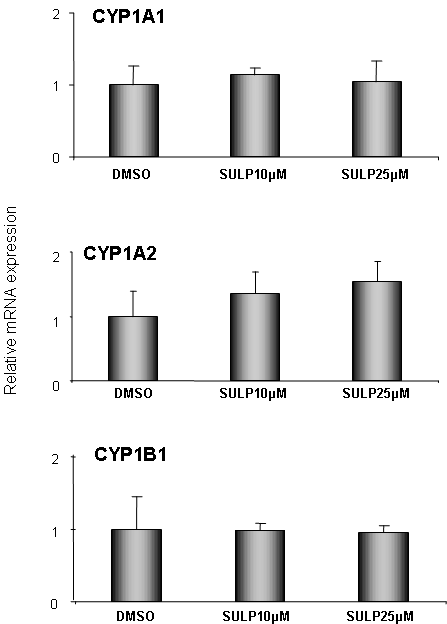

Supplement: S2 Fig — Alterations in CYP1A1, CYP1A2 and CYP1B1 relative mRNA expression following exposure of primary hepatocytes with either 10μM or 25μM sulpiride (SULP) for 24 hr. Control cells were treated with DMSO. (DOC) [file pone.0128708.s002.doc]
