# Supplementary material for: Dopamine D2-Receptor Antagonists Down-Regulate CYP1A1/2 and CYP1B1 in the Rat Liver
Source: PLoS One. 2015 Oct 14;10(10):e0128708. doi: 10.1371/journal.pone.0128708 (PMC4605514; doi:10.1371/journal.pone.0128708)

**S3 Figure.** **Sulpiride effect on constitutive and B[a]P-induced *HIF1a* mRNA expression in the rat liver.**


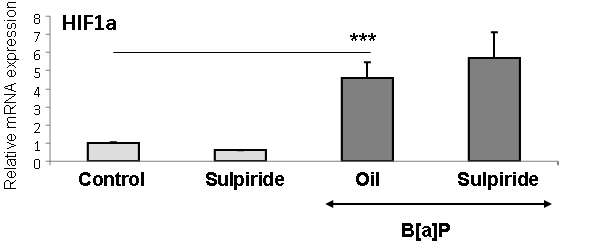

Supplement: S3 Fig — ***P<0.001. (DOC) [file pone.0128708.s003.doc]
