# Supplementary material for: Dopamine D2-Receptor Antagonists Down-Regulate CYP1A1/2 and CYP1B1 in the Rat Liver
Source: PLoS One. 2015 Oct 14;10(10):e0128708. doi: 10.1371/journal.pone.0128708 (PMC4605514; doi:10.1371/journal.pone.0128708)
Supplement: S1 Table — These primers were used for the quantitation of the mRNA levels of the genes tested using q-PCR. (DOC) [file pone.0128708.s004.doc]

**S1 Table. Nucleotide sequences of the primers used**

| **Gene** | **Primer sequence** |
| --- | --- |
| ***CYP1A1*** | F: 5΄-GTTCCCAAAGGTCTGAAGAG-3΄ |
|  | R: 5΄-CATATGGCACAGATGACATTGG-3΄ |
| ***CYP1A2*** | F: 5΄-TCGTACAGATGGCGTTCTCC-3΄ |
|  | R: 5΄-CGTCCCCATACTGCTGACTC-3΄ |
| ***CYP1B1*** | F: 5΄- ACCGCAAACTTCAGCAACTTC-3΄ |
|  | R: 5΄-GTACGGTGGTGACGGTTGTG-3΄ |
| ***AhR*** | F: 5΄-ATGGTCAGTCCTCAGGCGTACTA-3΄ |
|  | R: 5΄-AATGCTCGGACTCTGAAACTTGC-3΄ |
| ***ARNT*** | F: 5΄-AGCGACGGTCAGGGCTGGAT-3΄ |
|  | R: 5΄-CCCCTGGCCAGCCTCTGGAT-3΄ |
| ***AHRR*** | F: 5΄- GCCTGGTGCCCCATGGTTCC-3΄ |
|  | R: 5΄-CCCCTGGCGGTGAAGCACTG-3΄ |
| ***HSP90aa1*** | F: 5΄-GCTTCAGTGTCCCGGTGCGG-3΄ |
|  | R: 5΄-GCAGCTCCTTCCCCGAGTCC-3΄ |
| ***HIF1a*** | F: 5΄- AGCAATTCTCCAAGCCCTCC-3΄ |
|  | R: 5΄-TTCATCAGTGGTGGCAGTTG-3΄ |
